# Protocol for assisting frequency band definition and decoding neural dynamics using hierarchical clustering and multivariate pattern analysis

**DOI:** 10.1016/j.xpro.2025.103870

**Published:** 2025-06-03

**Authors:** Chengpeng Li, Isao Hasegawa, Hisashi Tanigawa

**Affiliations:** 1 Interdisciplinary Institute of Neuroscience and Technology, School of Brain Science and Brain Medicine, Zhejiang University, Hangzhou, China; 2 College of Biomedical Engineering and Instrument Science, Zhejiang University, Hangzhou, China; 3 MOE Frontier Science Center for Brain Science and Brain-Machine Integration, School of Brain Science and Brain Medicine, Zhejiang University, Hangzhou, China; 4 National Key Laboratory of Brain and Computer Intelligence, Zhejiang University, Hangzhou, China; 5 Department of Physiology, Niigata University School of Medical and Dental Sciences, Niigata, Japan

**Keywords:** Neuroscience, Cognitive Neuroscience, Biotechnology and bioengineering

## Abstract

Traditional fixed frequency band divisions often limit neural data analysis accuracy. Here, we present a protocol for assisting frequency band definition for multichannel neural data using macaque electrocorticography (ECoG) data. We describe steps for performing time-frequency analysis on preprocessed signals and applying hierarchical clustering to frequency power profiles to identify data-informed groupings. We then detail procedures for defining frequency bands guided by these clusters and using multivariate pattern analysis (MVPA) on the derived bands for functional validation via time-series decoding.

For complete details on the use and execution of this protocol, please refer to Tanigawa et al.^1^

## Highlights

• Steps for defining frequency bands via hierarchical clustering
• Procedures for time-frequency analysis and data clustering
• Instructions for MVPA validation of derived frequency bands
• Guidance on identifying functionally distinct neural sub-bands

## Abstract (editor highlights)

Publisher’s note: Undertaking any experimental protocol requires adherence to local institutional guidelines for laboratory safety and ethics.

## Graphical abstract

**Figure:**
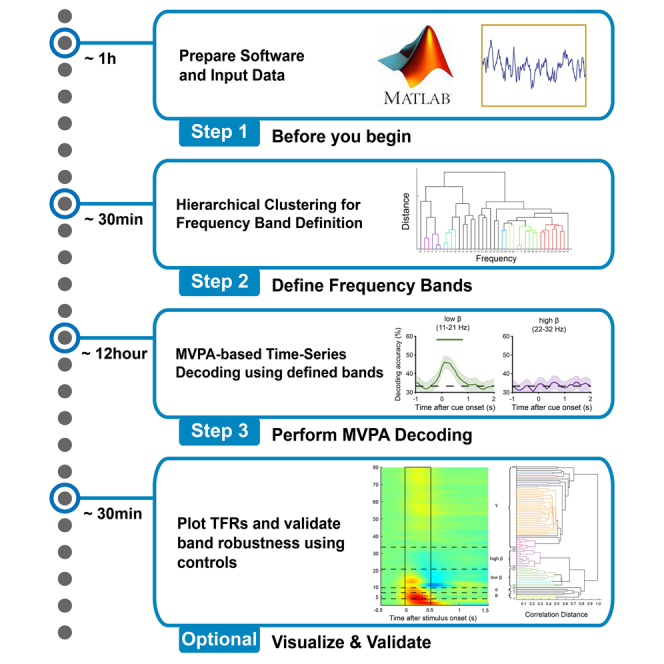

## Before you begin

Traditional methods for dividing the frequency spectrum in neural data analysis often have limitations. Conventional techniques typically use predefined bands such as delta (0.5–4 Hz), theta (4–8 Hz), alpha (8–13 Hz), beta (13–30 Hz), and gamma (30–100 Hz), based on historical and empirical observations. However, the definitions of these band ranges vary among researchers, leading to confusion and potential inconsistencies in data interpretation.^2^ While frequency bands are thought to be roughly conserved across mammalian species despite differences in brain size,^3^ non-negligible variability exists between individuals.^4^ Moreover, task conditions can influence frequency-specific effects. For example, effects traditionally associated with the alpha band may appear beyond conventional frequency ranges,^5^ or shifts in the gamma band frequency range may occur.^6^ These observations suggest that fixed band divisions may not adequately capture the complexity of neural oscillatory activity, potentially leading to a loss of important information and reduced accuracy in neural decoding.

To address these limitations, this protocol uses hierarchical clustering to inform the definition of frequency bands based on the inherent structure of the data. The protocol first derives a hierarchical cluster structure based on similarities within the data itself to overcome the constraints of conventional fixed band divisions. This resulting structure then guides subsequent manual refinement, enabling informed decisions about the final band boundaries. Leveraging clustering results to guide manual adjustments helps reveal novel and potentially more functionally relevant frequency bands that might be overlooked by conventional methods.

This protocol assists frequency band definition and evaluation in multielectrode neural oscillation data by combining hierarchical clustering with multivariate pattern analysis (MVPA)-based time-series decoding.^1^ Hierarchical clustering informs the definition of frequency bands, while MVPA-based time-series decoding evaluates their functional relevance and temporal dynamics. We anticipate that this protocol will be applicable across different individuals, brain regions, and experimental conditions, facilitating more detailed investigation and potentially providing valuable insights into neural oscillations.

### Institutional permissions

The electrocorticography (ECoG) data used as sample data in this protocol were obtained from two Japanese monkeys (Macaca fuscata). These monkeys were provided by NBRP "Japanese Monkeys" through the National BioResource Project of the MEXT, Japan. Some of these data were also used in our previous studies.^1^^,^^7^ The experimental protocol was approved by the Institutional Animal Care and Use Committee of Niigata University (Permission number 27-184-1). Furthermore, all animal procedures conformed to the Act on Welfare and Management of Animals in Japan, Fundamental Guidelines for Proper Conduct of Animal Experiment and Related Activities in Academic Research Institutions under the jurisdiction of the MEXT, Japan, and the National Institute of Health Guide for the Care and Use of Laboratory Animals.

### Data collection

This protocol is designed for analyzing neural oscillations from multichannel electrophysiological time series data. It is applicable to signals acquired using various methodologies, including non-invasive recordings like electroencephalography (EEG) and magnetoencephalography (MEG), as well as intracranial recordings such as ECoG and local field potential (LFP) recordings using penetrating multielectrode arrays like Utah arrays. While the analytical approach described herein can be generalized to various continuous time-series neural potential signals intended for oscillatory analysis, this protocol specifically demonstrates its application using ECoG data recorded from macaque monkeys. The subsequent analytical steps, however, operate on the time-frequency characteristics of the signals and are thus expected to be adaptable to data derived from various sensory modalities or cognitive tasks.

The sample data used in this protocol are local field potentials (LFPs) recorded from ECoG electrodes implanted on the cortical surface of macaque monkeys. Specifically, we use data from the prefrontal cortex (PFC) and inferior temporal cortex (ITC). The electrode configuration consists of 64 channels (8 × 8) for PFC and 128 channels for ITC (8 × 16), with an electrode spacing of 2.5 mm.

To demonstrate the versatility of the protocol, we selected neural signals from passive viewing tasks conducted on different dates. During these tasks, macaques passively observed visual stimuli composed of various color and shape combinations. LFPs were recorded simultaneously from high-density ECoG electrodes implanted in both the PFC and ITC during task performance.

For details about the experimental paradigm, including specific instructions, task sets, and timing, please refer to our previous research.^1^^,^^7^

***Note:*** The dataset used in this study is the same as that described in Tanigawa et al. (2022). No additional curation or manual trial selection was applied beyond standard preprocessing procedures. The preprocessing pipeline, including artifact removal and filtering, was performed using FieldTrip,^8^ ensuring consistency with previous studies.

### Pre-processing of data

**Timing: Several hours or more (depending on dataset size/resources)**

***Note:*** The following steps outline a general pre-processing pipeline. Adapt specific parameters to your data and research question. The estimated timing reflects computational time for automated steps. No additional manual curation was performed on the demonstration dataset beyond these steps.

1. Segment neural data.
  a. Segment raw neural signals into epochs based on the experimental task, typically time-locked to a specific event (e.g., onset of a cue stimulus).
  b. Define the time window for segmentation to capture the relevant neural activity.

***Note:*** In the sample data, a time window from 1.5 seconds before to 3.0 seconds after stimulus onset was used.

2. Apply band-pass filter.
  a. Apply a band-pass filter to the segmented data to focus the analysis on the desired frequency range.
  b. Determine the specific filtering range to encompass signal characteristics and the specific frequency bands under investigation.

***Note:*** In the sample data, a band-pass filter of 0.7–250 Hz was applied.

3. Downsample data (if applicable).
  a. Resample the data to a lower sampling frequency using an appropriate function (e.g., `ft_resampledata` in FieldTrip), ensuring the new sampling rate is adequate for the highest frequency of interest (Nyquist theorem).

***Note:*** In the sample data, the data was downsampled to 500 Hz after filtering. Consider downsampling to reduce computational load if appropriate for your analysis bandwidth.

4. Reduce noise and artifacts.
  a. Apply a method to reduce noise and artifact contamination (e.g., Independent Component Analysis (ICA)).

***Note:*** We applied ICA using the FieldTrip toolbox to the sample data. ICA separates statistically independent components, allowing removal of artifacts such as eye blinks and muscle activity.^9^ At the user’s discretion, other methods such as Signal Space Projection (SSP) or Artifact Subspace Reconstruction (ASR) could also be considered.^10^^,^^11^

5. Re-reference data (optional).
  a. Determine if re-referencing is necessary or beneficial for your data modality, setup, goals, and noise characteristics.

***Note:*** As EEG/ECoG signals represent potential differences, the reference choice significantly impacts signal representation. Selecting an appropriate strategy, or choosing not to re-reference, requires careful user consideration. For the ECoG sample data demonstrated here, Common Average Referencing (CAR) was applied for consistency with our prior work.^1^^,^^7^ CAR subtracts the average signal across selected channels from each channel to reduce global signals.^12^ However, CAR is presented only as the specific example used for this demonstration data and is just one option. Other strategies exist (e.g., specific electrode reference, bipolar derivation, Laplacian), and the optimal choice must be justified by the user for their specific data.

**Figure 1:**
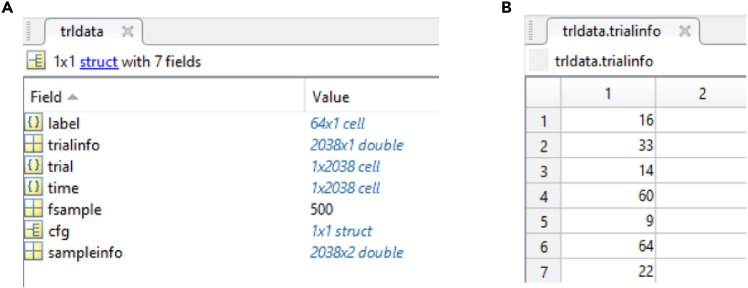
Required data structure for the protocol Data structure visualization showing the essential components of ‘trldata.mat’ required for the protocol. (A) The structure displays trial data, channel labels, time points, and trial information. (B) The ‘trialinfo’ field contains parameters for decoding analysis, here indicating the stimulus ID.

6. Format data for FieldTrip.
  a. Format the pre-processed data into the ‘trldata.mat’ structure compatible with the FieldTrip toolbox.
  b. Ensure the structure includes the following fields (Figure 1):
    i. trldata.trial: A cell array where each element is a matrix representing a trial (rows=channels, columns=time points).
    ii. trldata.label: A list of channel names corresponding to the recorded electrodes.
    iii. trldata.time: A cell array specifying the time points for each trial.
    iv. trldata.trialinfo: Metadata about the trials, such as stimulus IDs and condition.

***Optional:*** If starting from EEGLAB-formatted data, use the ‘eeglab2fieldtrip’ function in FieldTrip to convert your dataset. Confirm the resulting structure conforms to FieldTrip conventions.

### Software requirements and setup

**Timing: 30–60 min**

Follow these steps to install all required toolboxes in MATLAB.

7. Verify system and software requirements.
  a. Confirm your operating system compatibility.
    ***Note:*** This protocol has been tested on Windows 10 and Windows 11. We have also confirmed functionality on macOS, although specific modifications may be required (see troubleshooting, problem 3). While the core software components generally support Linux, we have not systematically validated the entire protocol pipeline across various Linux distributions. The notes regarding macOS may provide relevant insights for Linux users.
  b. Ensure sufficient RAM is available for your dataset size (at least 8GB recommended; 64GB used for sample data analysis). Larger datasets likely require more RAM.
  c. Use MATLAB version R2019b or later, as compatibility with earlier versions has not been verified.
  d. Install the required official MATLAB Add-On Toolboxes: Statistics and Machine Learning Toolbox, Signal Processing Toolbox, and Image Processing Toolbox.
8. Install FieldTrip toolbox.
  a. Download the FieldTrip toolbox from the official website (https://www.fieldtriptoolbox.org/download/).
    ***Note:*** Versions 20191213 and 20240110 are confirmed to work; later versions should be compatible, but avoid the latest unstable versions. Refer to the official FieldTrip documentation (https://www.fieldtriptoolbox.org/faq/installation/) for detailed installation instructions.
  b. Add FieldTrip to the MATLAB path using ‘ft_defaults’, following the official documentation to avoid conflicts (see troubleshooting, problem 4).
9. Install SVM solver (LibLinear and/or LIBSVM).
  a. Choose and install the appropriate SVM solver for your analysis. We recommend LibLinear^13^ for its efficiency with linear SVMs; LIBSVM^14^ is an alternative, especially if non-linear SVMs are needed, though generally slower for linear tasks.
  b. Download the chosen solver(s) from the official websites:
    i. LibLinear: https://www.csie.ntu.edu.tw/∼cjlin/liblinear/.
      ***Note:*** For LibLinear versions 2.48 (released January 5, 2025) and later on Windows, you must compile the MEX files manually. Instructions are typically in the README within the 'matlab' subfolder. Consider using earlier versions with pre-compiled Windows MEX files for convenience.
    ii. LIBSVM: https://www.csie.ntu.edu.tw/∼cjlin/libsvm/.
  c. Follow the installation instructions provided with the downloaded toolboxes.
  d. Compile MEX files if necessary (especially for macOS/Linux or newer LibLinear versions on Windows), referring to the solver’s README file for instructions (see also troubleshooting, problem 3).
10. Prepare other toolboxes and custom functions.
  a. Download the 'toolbox' folder (containing other necessary toolboxes and custom functions) from the designated repository (Zenodo: https://doi.org/10.5281/zenodo.14697222) to a local directory.
  b. Add the downloaded 'toolbox' folder, including all its subfolders, to the MATLAB path.
11. Prepare sample code and data.
  a. Download the 'sample_code' and 'sample_data' folders from the designated repository (Zenodo: https://doi.org/10.5281/zenodo.14697222).
    ***Note:*** The 'sample_data' folder contains 'trldata_odd.mat' (for clustering) and 'trldata_even.mat' (for decoding), derived from the original dataset to ensure independence.
  b. Place the downloaded folders in an accessible location for running the sample scripts.
    ***Note:*** The sample code is configured to use these specific files. Splitting data (e.g., odd/even trials) is one method to avoid circularity between band definition and decoding evaluation. Other valid splitting strategies exist depending on the experimental design.

## Key resources table

**Table:** 

REAGENT or RESOURCE	SOURCE	IDENTIFIER
**Experimental models: Organisms/strains**		

Japanese macaque (*Macaca fuscata*; 1 female used for sample data; age 7 years at the time of recording; weight 5.6 kg)	NBRP “Japanese Monkeys” through the National BioResource Project of the MEXT, Japan	https://nihonzaru.jp/aboutus_2_e.html

**Deposited data**		

Example datasets	This protocol	Zenodo: https://doi.org/10.5281/zenodo.14697222

**Software and algorithms**		

MATLAB (R2019b or later)	The MathWorks, Inc	RRID: SCR_001622 https://www.mathworks.com/products/matlab.html
MATLAB add-on toolboxes (Statistics and Machine Learning Toolbox, Signal Processing Toolbox, and Image Processing Toolbox)	The MathWorks, Inc	RRID: SCR_001622 https://www.mathworks.com/products/matlab.html
FieldTrip (fieldtrip-20191213 or later)	Open Source Oostenveld et al.^8^	RRID: SCR_004849 https://www.fieldtriptoolbox.org/
LIBLINEAR version 2.47	Open Source Fan et al.^13^	https://www.csie.ntu.edu.tw/∼cjlin/liblinear/
LIBSVM version 3.35	Open Source Chang and Lin^14^	https://www.csie.ntu.edu.tw/∼cjlin/libsvm/
Custom MATLAB scripts and functions	This protocol	Zenodo: https://doi.org/10.5281/zenodo.14697222

## Step-by-step method details

### Time-frequency analysis for hierarchical clustering

**Timing: 30–60 min**

This section describes the process of generating the Time-Frequency Representation (TFR) of the pre-processed data using the FieldTrip toolbox. This is performed for the subsequent hierarchical clustering analysis. The TFR provides a representation of the data’s power in both the time and frequency domains. The MATLAB script ‘P1_Averaging_TFR_div00.m’ is used to perform the steps described below. Running this script will create the `TFR_trldata` structure that will be used as input for the next stage of analysis.

***Note:*** The sample dataset (`trldata_odd.mat`) used in the example code can be found in the ‘sample_data’ folder, and the corresponding script (‘P1_Averaging_TFR_div00.m’) is located in the ‘sample_code’ folder within the designated repository. `trldata_odd.mat` contains only the odd-numbered trials from the original dataset, prepared specifically to ensure independence between the data used for clustering and decoding.

1. Configure parameters and file paths for TFR analysis.
  a. Before running the script ‘P1_Averaging_TFR_div00.m’, adjust the parameters within the script that control data import and TFR analysis.
    ***Note:*** The key parameters to set are shown in the example code block below.
    % Define input folder
    input_path = 'D:\sample_data';
    trldata_name = 'trldata_odd.mat';
    % Define channels of interest and suffix for saving the TFR data
    slct_channel = {'all'};
    savename_postfix='_odd';
    % Define time of interest
    toi = −1.0:0.05:2.0;
    % Define frequencies of interest
    foi = 1:1:80;
    % Define parameters for TFR calculation
    TFRMethod = 1;
    length_timwin = 0.5;
    flgTFRHz = 1;
    flgFbAveraging = 0;
  b. Set the `input_path` variable to specify the path to the folder where the input data file (e.g., `trldata.mat`) is located (e.g., 'D:\sample_data').
  c. Define the `trldata_name` variable as the name of the input MATLAB data file (e.g., 'trldata_odd.mat').
    ***Note:*** The sample code uses `trldata_odd.mat`, containing only odd-numbered trials, for this clustering-related TFR calculation to ensure data independence.
  d. Define the `slct_channel` variable to specify the channels to be analyzed (e.g., {'all'}).
  e. Set the `savename_postfix` variable to append a user-specified suffix to the output filename (e.g., '_odd') to help identify the specific analysis parameters used.
  f. Define the `toi` variable for the time range of interest (in seconds) for the TFR analysis (e.g., −1.0:0.05:2.0 for −1.0 s to 2.0 s in 0.05 s steps).
  g. Define the `foi` variable for the frequencies of interest (in Hz) for the TFR analysis (e.g., 1:1:80 for 1 Hz to 80 Hz).
  h. Select the TFR calculation method by setting the `TFRMethod` variable to 1 for ‘wavelet’ (wavelet transform, used in this protocol) or 2 for ‘mtmconvol’ (multitaper method with convolution).
    ***Note:*** This protocol utilizes the ‘wavelet’ method, which employs Morlet wavelets. Our implementation sets the wavelet width (`cfg.width`) based on frequency and `length_timwin` (see ‘P1_Averaging_TFR_div00.m’ script for details). This generally provides adaptive time-frequency resolution and is the default, following our prior related work. The ‘mtmconvol’ method, an alternative, implements a Short-Time Fourier Transform (STFT) with a Hanning taper and a fixed time window (`length_timwin`), yielding fixed time-frequency resolution. The primary impact of choosing between these methods is the time-frequency resolution trade-off.
  i. Specify the `length_timwin` variable for the length of the time window (in seconds) used for the analysis (e.g., 0.5).
    ***Note:*** For the ‘mtmconvol’ method, `length_timwin` determines the sliding window length. For the wavelet method, it represents the length of the time window, typically determined by the lowest frequency of interest. If `length_timwin` is set too short, it may result in insufficient calculation of low-frequency information; conversely, if set too long, it may reduce the time resolution of high-frequency information. We use 0.5 seconds as the default.
  j. Set the `flgTFRHz` variable to 1 to normalize powers by 1/Hz (default), or 0 otherwise.
    ***Note:*** Applying 1/Hz power normalization (`flgTFRHz`=1) is a common technique to compensate for the characteristic 1/f power scaling (higher power at lower frequencies) in electrophysiological data. This normalization emphasizes power variations relative to the 1/f trend. Other approaches, such as baseline normalization or using raw power, may be considered by modifying the TFR calculation step.
  k. Set the `flgFbAveraging` variable to 0 to calculate power for every frequency (e.g., every 1 Hz) for the hierarchical clustering analysis.
    ***Note:*** Set to 1 to define specific frequency bands for averaging when performing TFR analysis for neural decoding (described in 'time-frequency analysis for neural decoding' section). If `flgFbAveraging` is set to 1, define the frequency bands in the `freqband` variable.
    **CRITICAL:** Ensure all parameters, especially `toi`, `foi`, `TFRMethod`, and `length_timwin`, are set appropriately for your specific data and research question. Incorrect settings will lead to invalid TFR results, impacting subsequent clustering analysis. Using the correct input dataset (e.g., `trldata_odd.mat` for clustering) is essential for ensuring independence from the decoding dataset.
2. Run the script and save the TFR data.
  a. Once the parameters are configured, run the script ‘P1_Averaging_TFR_div00.m’.

***Note:*** This script utilizes the parameters you have set, imports the specified `trldata_name` file (e.g., `trldata_odd.mat`), performs the time-frequency analysis using the `ft_freqanalysis` function from FieldTrip, and saves the results in a new MATLAB structure named `TFR_trldata`. The `TFR_trldata` is saved in the ‘processed’ folder within the `input_path`. The filename will automatically include ‘div00’ to indicate no frequency band averaging, followed by the user-defined `savename_postfix`.

**Pause point:** The analysis can be safely paused here after the TFR data for clustering (e.g., ‘TFR_trldata_div00_odd.mat‘) has been successfully generated and saved.

### Hierarchical clustering of time-frequency power

**Timing: 5–15 min**

This section describes the process of performing hierarchical clustering on the time-frequency power data (`TFR_trldata`) generated in Step 2. The goal is to identify clusters of frequencies that exhibit similar temporal power profiles. The MATLAB script ‘P2_Hclust_freq_bands.m’ is used to perform the steps described below. Running this script will generate a dendrogram visualizing the hierarchical clustering of frequency bands and other related variables.

3. Configure parameters and file paths for hierarchical clustering.
  a. Before running the script ‘P2_Hclust_freq_bands.m’, adjust the parameters within the script that control the hierarchical clustering analysis.
    ***Note:*** The key parameters to set are shown in the example code block below.
    % Input data path and file names
    flgLoadTFRdata = 1;
    input_path = 'D:\sample_data';
    TFR_trldata_name = 'TFR_trldata_div00_odd';
    save_name = 'cluster_results_div00_odd';
    % Clustering options
    rpt_ave = 1;
    cluster_target = 1;
    dB_conversion = 1;
    if dB_conversion == 1
     baseline_timerange = [6:16];
    end
    linkage_method = 1;
    cutoff_ratio = 0.75;
    freq_range = 4:80;
  b. Set `flgLoadTFRdata` to 1 to load the `TFR_trldata` file generated in Step 2, or 0 to skip loading for testing purposes.
  c. Specify `input_path` as the path to the folder containing the ‘processed’ folder (where `TFR_trldata.mat` from Step 2 is located, e.g., 'D:\sample_data').
  d. Define `TFR_trldata_name` as the name of the `TFR_trldata` file generated in Step 2 (e.g., 'TFR_trldata_div00_odd.mat').
  e. Assign `save_name` as a user-defined name for saving the results of the hierarchical clustering analysis (e.g., 'cluster_results_div00_odd').
  f. Set `rpt_ave` to 1 to average power across trials before clustering (default), or 0 to perform clustering on each individual trial.
  g. Set `cluster_target` to 1 to cluster frequencies based on the similarity of their time-power profiles (default), or 2 to cluster channels.
  h. Set `dB_conversion` to 1 to perform decibel (dB) conversion on the power data relative to a baseline period before clustering (default); set to 0 if dB conversion should not be applied.
    ***Note:*** The purpose of dB conversion (10∗log10(power/baseline)) is to normalize power with respect to a baseline, emphasizing relative power changes. This is often suitable for analyzing stimulus-evoked responses, as demonstrated with the sample data in this protocol. However, for analyses without a clear baseline (e.g., resting-state) or where absolute power is desired, users should set this parameter to 0.
  i. Define baseline_timerange (e.g., [6:16]) as the time points (indices) used for dB conversion if `dB_conversion` is set to 1.
    ***Note:*** For example, if the sampling interval is 0.05 s, and the desired baseline is −0.8 s to −0.2 s, set this to baseline_timerange = [6:16];.
  j. Select `linkage_method` for the hierarchical clustering algorithm: 1 for 'average' linkage (default) or 2 for 'complete' linkage (farthest neighbor).
    ***Note:*** The `linkage_method` determines how the dissimilarity between two merging clusters is calculated at each step of the agglomerative clustering process, based on the pairwise distances between the elements within them. 'Average' linkage calculates the average distance between all pairs of objects, one in each cluster, and is often considered robust to noise, tending to produce balanced clusters. 'Complete' linkage uses the maximum distance between any object in one cluster and any object in the other, tending to find compact, spherical clusters but can be sensitive to outliers. Other methods like 'single' or 'Ward's' exist and can be implemented by modifying the call to the linkage function in ‘P2_Hclust_freq_bands.m’.
  k. Specify `cutoff_ratio` (e.g., 0.75) as a ratio (typically 0-1) used for the calculation of the cluster cutoff threshold (`coph`).
    ***Note:*** Lower values generally lead to finer clusters, higher values to coarser ones. Adjust based on evaluation criteria (see troubleshooting, problem 5).
  l. Define `freq_range` (e.g., 4:80) as the frequency range (in Hz) to be used for clustering.
    **CRITICAL:** Parameter settings for clustering, particularly `dB_conversion`, `baseline_timerange` (if applicable), `linkage_method`, and `cutoff_ratio`, significantly influence the resulting dendrogram structure. Carefully consider and justify these choices based on your data properties and analysis goals (see troubleshooting, problem 5).
4. Run the script.
  a. After configuring the parameters, run the script ‘P2_Hclust_freq_bands.m’.
    ***Note:*** This script performs several key steps: it loads the `TFR_trldata` generated in Step 1, optionally normalizes power values based on the selected method (`dB_conversion`) and `baseline_timerange` (if applicable), calculates the distance between frequency (or channel, depending on `cluster_target`) power profiles using a correlation distance metric, performs hierarchical clustering using the `linkage` function with the chosen `linkage_method`, determines the optimal leaf order for the dendrogram using the `optimalleaforder` function, visualizes the resulting dendrogram, and identifies clusters based on a specified threshold (derived from `cutoff_ratio`) and the `cluster` function.
  b. The script saves the results of the hierarchical clustering, including the dendrogram and other associated variables, in the ‘hclust’ folder within the ‘results’ folder inside the `input_path`.
    **Pause point:** The analysis can be paused after the hierarchical clustering results (including the dendrogram information and automatically identified bands) are saved.
5. Interpret the generated dendrogram.
  a. Examine the visualized dendrogram to understand how frequencies (or channels) are grouped based on the similarity of their power time courses.
    ***Note:*** The script generates a dendrogram where the height of the branches represents the distance between clusters. Frequencies merging at smaller distances (further right in the default left-oriented plot) are considered more similar. The `clusterTree` variable, created by the `linkage` function, stores the hierarchical clustering information. The `leafOrder` variable, from `optimalleaforder`, contains the optimal ordering of leaves. The estimated cophenetic correlation coefficient (`coph` value calculated by `cophenet`) is also available in the workspace for reference, indicating how well the dendrogram preserves original dissimilarities.
  b. Understand the color coding of the dendrogram branches.
    ***Note:*** The dendrogram function in MATLAB automatically assigns colors to different branches based on the cluster hierarchy and the specified `ColorThreshold` parameter (default 0.5, adjustable by the user). It is crucial to understand that these automatically assigned colors serve only as visual aids to differentiate sub-trees and do not necessarily represent the final, meaningful frequency bands for analysis.
    **CRITICAL:** The interpretation of the dendrogram forms the basis for frequency band definition. Misinterpretation can lead to suboptimal band selection for subsequent analyses.
6. Define frequency bands based on the dendrogram.
  ***Note:*** Use the generated dendrogram to define frequency bands for further analysis. Two complementary approaches, manual selection and automatic subdivision, are available and can be used in combination.
  a. Perform manual selection of frequency bands.
    i. Visually inspect the dendrogram and identify clusters of frequencies grouped at a desired level of similarity (distance).
    ii. Use conventional frequency band definitions (e.g., theta: 4–8 Hz, alpha: 8–13 Hz) as a reference point.
    iii. Identify clusters in the dendrogram that overlap with these conventional bands.
    iv. Adopt the conventional definition if a cluster aligns well with it.
    v. Define new frequency bands based on the dendrogram’s branching pattern if a cluster spans multiple conventional bands or shows a finer structure.
  b. Utilize automatic subdivision as a starting point for band definition.
    ***Note:*** The script automatically identifies potential frequency bands based on the cluster cutoff threshold (`coph` value used in the `cluster` function, derived from `cutoff_ratio`). The `cluster` function extracts these clusters from the `clusterTree` structure based on the inconsistency coefficient (provided in the `inconsistent_data` variable). The `new_bands` variable, saved in the ‘hclust’ folder, will contain the start and end frequencies of these automatically identified bands. These automatically identified bands can serve as a starting point for manual refinement; you might need to merge or further subdivide these bands based on visual inspection of the dendrogram and your research question (e.g., merging narrow gamma sub-bands into a single gamma band).
  c. Finalize the new frequency bands to be used in subsequent parts of the protocol.
    **CRITICAL:** The final definition of frequency bands based on the dendrogram requires careful interpretation and justification. Ensure the selected bands are consistent with the clustering structure and, ideally, validated for functional relevance using independent data (as described in Steps 9–12 and troubleshooting, problem 5). The chosen bands directly impact the subsequent decoding analysis.

### Time-frequency analysis for neural decoding

**Timing: 30–60 min**

This section describes the process of generating a new Time-Frequency Representation (TFR) of the pre-processed data, with power averaged across specific frequency bands. This band-averaged TFR will be used for the neural decoding analysis. The MATLAB script ‘P3_Averaging_TFR_div01.m’ is used. This script is nearly identical to ‘P1_Averaging_TFR_div00.m’ (used in Step 1), but with crucial differences in the input data file and the parameters `flgFbAveraging` and `freqband`, as highlighted below.

***Note:*** To ensure independence between the data used for clustering (described in the 'hierarchical clustering of time-frequency power' section) and decoding, this step must use the dataset reserved for decoding (e.g., `trldata_even.mat` in the sample code).

7. Configure parameters and file paths for band-averaged TFR analysis.
  a. Before running the script ‘P3_Averaging_TFR_div01.m’, adjust the parameters within the script. For parameters common with Step 1, refer to the descriptions in Step 1 and adjust them as necessary for this analysis stage.
    ***Note:*** The key parameters that differ or require specific attention for this step are shown in the example code block below.
    trldata_name = 'trldata_even.mat';
    savename_postfix='_even';
    flgFbAveraging = 1;
    if flgFbAveraging
     freqband = {4:7,8:10,11:21,22:32,33:80}
    end
  b. Set the `trldata_name` variable to the name of the independent input MATLAB data file reserved for decoding (e.g., 'trldata_even.mat').
  c. Set the `savename_postfix` variable to a unique suffix for the output filename of this analysis stage (e.g., '_even').
  d. Set the `flgFbAveraging` variable to 1. This enables the averaging of power across the specific frequency bands defined in the next step.
  e. Define the `freqband` variable as a cell array specifying the frequency bands for averaging. Each cell must contain a vector defining the start and end frequencies of a band.
    ***Note:*** The `freqband` variable should be defined based on the frequency bands identified in Step 6 (Define frequency bands based on the dendrogram). The example bands shown in the code block ({4:7, 8:10, 11:21, 22:32, 33:80}) are illustrative and should be replaced with your actual derived or chosen bands.
    **CRITICAL:** Ensure `flgFbAveraging` is set to 1 and the `freqband` variable accurately reflects the frequency bands defined in Step 6. Using the correct independent dataset (e.g., `trldata_even.mat` specified in `trldata_name`) is crucial to avoid circularity and ensure a valid evaluation of the defined frequency bands.
8. Run the script and save the band-averaged TFR data.
  a. Once the parameters are configured, run the script ‘P3_Averaging_TFR_div01.m’.

***Note:*** This script performs time-frequency analysis using `ft_freqanalysis` (similar to the process in Step 2) and then averages the power spectrum across the frequency bins specified in each cell of the `freqband` variable. The new `TFR_trldata` structure is saved in the ‘processed’ folder within the `input_path`. The filename will automatically include a unique identifier (e.g., ‘div01’, depending on script versioning) to indicate that frequency band averaging has been performed, followed by the user-defined `savename_postfix` (e.g., resulting in a file like ‘TFR_trldata_div01_even.mat’).

**Pause point:** The analysis can be paused once the band-averaged TFR data for decoding (e.g., ‘TFR_trldata_div01_even.mat’) is generated and saved.

### Multivariate pattern analysis for time-series neural decoding

**Timing: Approximately several hours to several days for the bootstrap test with 1,000 iterations**

This section outlines the steps for performing MVPA-time-series decoding as demonstrated within this protocol’s framework using specific software (e.g., LibLinear/LIBSVM) on the time-frequency representation data (`TFR_trldata`) generated in Step 8. The goal is to decode the information represented in distinct frequency bands using an n-fold cross-validation approach and, optionally, to assess the statistical significance of the decoding results using a bootstrap resampling. This protocol performs multiclass classification, where each class is defined by the `class_stim` parameter. The MATLAB script ‘P4_TC_decoding.m’ provides an example implementation of these steps.

9. Configure parameters and file paths for MVPA time-series decoding.
  a. Before running the script ‘P4_TC_decoding.m’, adjust the parameters within the script that control the MVPA.
    ***Note:*** The key parameters to set are shown in the example code block below.
    % Define input folder and TFR data file
    input_path = 'D:\sample_data';
    TFR_trldata_name = 'TFR_trldata_div01_even';
    output_postfix = '_even';
    % Define time points for training and testing
    slct_time_point.training = [1:2:61];
    slct_time_point.test = [1:2:61];
    % Cross-validation and statistical testing parameters
    num_fold = 10;
    flg_bootstrap = 1;
    ntimes_iter = 1000;
    % Data selection parameters
    num_channel = 64;
    slct_freqb = [1:5];
    % Define classes for decoding
    class_stim(1,:) = [1:12 37:48];
    class_stim(2,:) = [13:24 49:60];
    class_stim(3,:) = [25:36 61:72];
    % SVM solver parameters
    svm_solver = 2;
    decoding_option = '-s 2 -q -c 0.001';
  b. Set the `input_path` variable to specify the path to the folder where the input data file (`TFR_trldata`) from Step 8 is located (e.g., 'D:\sample_data').
  c. Define the `TFR_trldata_name` variable as the name of the `TFR_trldata` file generated in Step 8 (e.g., 'TFR_trldata_div01_even.mat').
  d. Set the `output_postfix` variable to a user-defined string that will be appended to the output filename (e.g., '_even').
  e. Define the `slct_time_point.training` vector specifying the indices of the time points to be used for training the decoder (e.g., [1:2:61]).
  f. Define the `slct_time_point.test` vector specifying the indices of the time points to be used for testing the decoder.
    ***Note:*** This should typically be the same as `slct_time_point.training`. In the example, every other time point is selected to reduce computation time. If users are analyzing their own complete dataset, they can include all relevant time points (e.g., [1:61]). Remember that the time points are indices, not actual time values; refer to the `TFR_trldata.time` variable for actual time values.
  g. Set the `num_fold` variable for the number of folds to use for cross-validation (e.g., 10).
  h. Set the `flg_bootstrap` variable to 1 to perform bootstrap resampling for statistical testing (default), or 0 to use a binomial test.
    ***Note:*** In this protocol, bootstrap resampling is employed as the default method (`flg_bootstrap` = 1) for evaluating the statistical significance of decoding accuracy. This directly follows the procedure validated and adopted in our related research, 1 where comprehensive implementation details are described. In summary, within each cross-validation fold, this method involves creating bootstrap training datasets by repeatedly (e.g., `ntimes_iter` times) resampling the original training trials with replacement. A classifier is trained on each bootstrap dataset and then evaluated on the original held-out test dataset for that fold. This generates a distribution of decoding accuracies under the null hypothesis, allowing for the calculation of a *p*-value by comparing this distribution to the theoretical chance level. Subsequently, FDR correction is applied across time points. Using bootstrap resampling to establish the statistical significance of decoding performance is a well-established practice in MVPA studies within neuroscience.^15^^,^^16^ Permutation tests (e.g., shuffling class labels before training) are another widely accepted and powerful approach for non-parametric significance testing in MVPA. Users who prefer permutation-based methods or other statistical techniques would need to modify the provided analysis scripts accordingly. Although an option for a binomial test is also provided (`flg_bootstrap` = 0), its use for analysis is not recommended as it is known to be inappropriate for assessing classifier performance involving cross-validation, potentially yielding biased significance estimates.^17^ This option is included solely for users who wish to rapidly check code execution.
  i. Set the `ntimes_iter` variable for the number of bootstrap iterations to perform if `flg_bootstrap` is 1 (e.g., 1000 or more).
  j. Define the `num_channel` variable as the number of channels to use in the decoding analysis (e.g., 64).
  k. Define the `slct_freqb` vector specifying the indices of the frequency bands (defined in Step 6) to be used for decoding (e.g., [1:5]).
  l. Define the `class_stim` matrix for decoding. Each row represents a class, and the values in each row correspond to the trial numbers associated with that class, as indicated in `TFR_trldata.trialinfo`.
    ***Note:*** In the provided example, class 1 represents the experimental condition where a red visual stimulus was presented, class 2 represents green, and class 3 represents blue. Modify this according to your experimental design.
  m. Set the `svm_solver` variable to specify the SVM solver: 1 for LIBSVM, 2 for LibLinear.
  n. Define the `decoding_option` string containing the options for the chosen SVM solver.
    ***Note:*** These options are specific to the chosen solver (LIBSVM or LibLinear). Refer to the documentation of the respective solver for more details. The example `-s 2 -q -c 0.001′ is for LibLinear. Since the optimal option depends on various factors, such as experimental conditions and data types, users should select the optimal option.
    **CRITICAL:** Correct configuration of MVPA parameters, including cross-validation folds (`num_fold`), statistical testing method (`flg_bootstrap`, `ntimes_iter`), selected frequency bands (`slct_freqb`), class definitions (`class_stim`), and SVM solver options (`svm_solver`, `decoding_option`), is essential for obtaining meaningful and statistically sound decoding results. Verify that the correct band-averaged TFR file (specified by `TFR_trldata_name`) is used as input.
    ***Note:*** While this protocol demonstrates decoding using LibLinear/LIBSVM due to their computational efficiency for this type of analysis, other machine learning classifiers suitable for MVPA could potentially be substituted at this stage. However, implementing alternative classifiers would require modifying the relevant sections of the provided MATLAB scripts.
10. Run the script and save the decoding results.
  a. Once the parameters are configured, run the script ‘P4_TC_decoding.m’.

***Note:*** This script loads the `TFR_trldata` generated in Step 8, selects the data based on the specified channels, frequency bands, and time points, and prepares the data for decoding by arranging it into feature vectors and labels. It then performs cross-validation using the specified number of folds (`num_fold`). For each time point, frequency band, and fold, the script trains an SVM classifier on the training data and tests the trained classifier on the test data, calculating the decoding accuracy. It also performs bootstrap resampling or a binomial test (based on `flg_bootstrap`) to estimate the statistical significance of the decoding results, and averages the decoding accuracy across folds and bootstrap iterations. The decoding results are saved as a MATLAB structure named 'decoding_results' within the ‘decoding’ folder inside the `input_path`. The ‘decoding_results’ structure contains the following key variables: `decoding_results.rate_ave` (average decoding accuracy matrix), `decoding_results.rate_ave_pw` (average decoding accuracy per class matrix).

**Pause point:** The MVPA decoding analysis can be time-consuming. The process can be paused after the `decoding_results` structure is saved.

### Visualizing and saving decoding accuracy time courses

**Timing: 5–10 min**

This section describes how to visualize the time-course decoding accuracy results generated in Step 10 using the provided MATLAB script ‘P5_TC_Plot.m’. This script loads the `decoding_results` structure and generates plots of the decoding accuracy over time, optionally including statistical significance indicators derived from the bootstrapping test. It also allows for customization of various plot parameters and saving the generated figures in different formats.

11. Configure parameters and paths for visualizing decoding accuracy.
  a. Before running the script ‘P5_TC_Plot.m’, adjust the parameters within the script that control the analysis and plotting.
    ***Note:*** The key parameters to set are shown in the example code block below.
    % Define input folder and decoding results file
    input_path = 'D:\sample_data';
    input_decoding_name = 'decd_fb15_even';
    output_plots_name = 'decd_fb15_even';
    % Define plot axis limits
    x_lim = [-1.0, 2.0];
    y_lim = [30 60];
    % Plotting options
    slct_freqb = [1:5];
    flag_line_signif = 1;
    % Statistical parameters
    flg_bootstrap = 1; % Should match the setting in Step 9g
    alpha_p = 0.05;
    side_for_stat = 1;
    flg_fdr = 1;
    % Figure saving options
    flg_one_figure = 0;
    flgSavePDF = 1;
    flgSaveEPS = 1;
    flgSavePNG = 1;
  b. Set the `input_path` variable to the location where the ‘decoding’ folder (containing the decoding results file generated in Step 10) is located.
  c. Define the `input_decoding_name` variable as the name of the decoding results file to be loaded.
  d. Set the `output_plots_name` variable to a user-defined prefix for the saved plots.
  e. Define the `x_lim` variable as a two-element vector specifying the x-axis limits for the plot (time in seconds).
  f. Define the `y_lim` variable as a two-element vector specifying the y-axis limits for the plot (decoding accuracy in %).
  g. Define the `slct_freqb` vector specifying the indices of the frequency bands (defined in Step 6) to be plotted.
  h. Set the `flag_line_signif` variable to 1 to draw significance lines on the plot, or 0 to omit them.
    ***Note:*** If `flg_bootstrap` (see sub-step h) was set to 1 during decoding (Step 9g), significant time points are determined based on the bootstrapped *p*-values and are indicated by horizontal lines above the plot. Additionally, when `flg_bootstrap` is 1, error bars representing the (100 - `alpha_p` ∗ 100)% confidence interval (e.g., 95% confidence interval for `alpha_p` = 0.05) are added to the plot. If `flg_bootstrap` was set to 0, significance is determined using the p-values from the binomial test.
  i. Ensure the `flg_bootstrap` variable is set consistently with the method used in Step 9g (typically 1 to use the bootstrap test for significance testing).
  j. Set the `alpha_p` variable for the significance level (alpha) for statistical testing (e.g., 0.05 for p < 0.05).
  k. Set the `side_for_stat` variable to specify a one-sided (1, default) or two-sided (2) test.
  l. Set the `flg_fdr` variable to 1 to apply False Discovery Rate (FDR) correction for multiple comparisons, or 0 to not apply it.
  m. Set the `flg_one_figure` variable to 1 to plot multiple time series on a single figure, or 0 to plot each time series in a separate figure (default).
  n. Set `flgSavePDF`, `flgSaveEPS`, and `flgSavePNG` variables to 1 to save the plots in the respective formats, or 0 to not save.
    **CRITICAL:** Ensure that the statistical parameters (`flg_bootstrap`, `alpha_p`, `side_for_stat`, `flg_fdr`) are set consistently with the analysis performed in Step 9 and Step 10 to accurately represent the statistical significance of the decoding results. The `input_decoding_name` must point to the correct output file from Step 10.
12. Run the script to visualize and save decoding accuracy plots.
  a. Once the parameters are configured, run the script ‘P5_TC_Plot.m’.

***Note:*** This script loads the specified `decoding_results` structure. If `flg_bootstrap` is set to 1, it performs a bootstrap test (or uses pre-calculated bootstrap results if available within the structure from Step 10). It then generates plots of decoding accuracy over time based on the configured parameters, including options for error bars, significance indicators, and displaying multiple lines on a single plot. Finally, it saves the generated figures in the specified formats in the ‘plots’ folder within the results folder inside the `input_path`.

**Pause point:** This step marks the end of the core data analysis and visualization part of the protocol. Results are generated and saved.

### Visualizing TFR

**Timing: 5–10 min**

This section provides an optional guide for visualizing the Time-Frequency Representation (TFR) data generated in Step 2 (the TFR data used for hierarchical clustering) using the FieldTrip function `ft_singleplotTFR`. This step can be helpful for exploring the data, verifying the parameters used during TFR calculation, and providing supplementary visual information to aid in the interpretation of the hierarchical clustering results or decoding analysis.

13. Configure parameters and paths for TFR visualization.
  a. Before running the script ‘P6_TFR_plot.m’, adjust the parameters within the script that control the visualization.
    ***Note:*** The key parameters to set are shown in the example code block below.
    % Flag to load TFR data
    flgLoadTFRdata = 1;
    % Define input folder and TFR data file name
    input_path = 'D:\sample_data';
    TFR_trldata_name = TFR_trldata_div00_odd'; % TFR data from Step 2
  b. Set the `flgLoadTFRdata` variable to 1 to load the `TFR_trldata` file generated in Step 2. Set to 0 to skip loading for testing purposes.
  c. Set the `input_path` variable to specify the path to the folder containing the ‘processed’ folder (where `TFR_trldata.mat` from Step 2 is located, e.g., 'D:\sample_data').
  d. Define the `TFR_trldata_name` variable as the name of the `TFR_trldata` file generated in Step 2 (e.g., 'TFR_trldata_div00_odd.mat').
14. Run the script to display the TFR plot.
  a. Once the parameters are configured, run the script ‘P6_TFR_plot.m’.

***Note:*** This script loads the specified `TFR_trldata` structure and displays a time-frequency plot using the `ft_singleplotTFR` function from the FieldTrip toolbox based on the configured parameters. This visualization can be useful for inspecting the overall time-frequency characteristics of the data used for clustering.

### Validation using temporally shuffled data

**Timing: Approx. 10–20 min per dataset (depending on dataset size) for data generation**

**Timing: Several hours to days (depending on dataset size and number of bootstrap iterations for decoding) for re-analysis (Steps 1–12)**

To ensure that the patterns identified by the clustering and decoding analyses on the original data reflect genuine neural dynamics rather than arising spuriously from noise or statistical properties inherent in the time series, this optional control analysis using temporally shuffled data is recommended. This procedure serves as a sanity check, addressing the possibility that data-driven methods like hierarchical clustering might find structure in data lacking true temporal dependencies.

15. Generate temporally shuffled datasets.
  ***Note:*** This step creates control datasets where the temporal structure within each channel and trial is destroyed, while preserving the overall power distribution within that channel/trial segment. Use the provided MATLAB script ‘P7_Temporal_Shuffle.m’.
  a. Prepare two separate shuffled datasets for independent validation of clustering and decoding:
    i. Generate `trldata_odd_shuffled.mat` by providing `trldata_odd.mat` (used for original clustering in Steps 3–6) as input to the script.
      input_filename = 'trldata_odd.mat';
    ii. Generate `trldata_even_shuffled.mat` by providing `trldata_even.mat` (used for original decoding in Steps 9–10) as input to the script.
      input_filename = 'trldata_even.mat';
      **CRITICAL:** Ensure the temporal shuffling procedure correctly randomizes time points within each trial and channel independently while preserving all other data structures and metadata. Incorrect shuffling will invalidate the control analysis.
      ***Note:*** The script randomizes the order of time points independently for each channel within each trial. All metadata (channel labels, trial information, time vectors) are preserved from the original data structure.
16. Re-run analysis pipeline on shuffled data.
  a. Apply the core analysis pipeline (Steps 1–12) to the shuffled datasets generated in Step 15.
    ***Note:*** Use the same parameters as the original analysis to allow for direct comparison.
  b. Clustering analysis control:
    i. Execute the 'Time-frequency analysis for hierarchical clustering' (Steps 1–2) using `trldata_odd_shuffled.mat` as the input data. Maintain all parameters identical to those used for the original analysis.
    ii. Execute the 'hierarchical clustering of time-frequency power' analysis (Steps 3–6) using the TFR results derived from `trldata_odd_shuffled.mat`.
  c. Decoding analysis control:
    i. Execute the 'time-frequency analysis for neural decoding' (Steps 7–8) using `trldata_even_shuffled.mat` as input. Define the frequency bands (`freqband` variable) for averaging by manually inspecting the dendrogram generated from the shuffled data in Step 16.a.ii.
      ***Note:*** Select cluster boundaries that appear to most closely approximate conventional frequency bands (e.g., theta, alpha, beta, gamma ranges) within the (likely unstructured) dendrogram. Use these manually selected, conventional-approximating bands for TFR calculation.
    ii. Execute the 'multivariate pattern analysis for time-series neural decoding' (Steps 9–10) using the TFR results derived from `trldata_even_shuffled.mat`.
    iii. Execute the 'visualizing and saving decoding accuracy time courses' analysis (Steps 11–12) using the decoding results from the shuffled data.
      **CRITICAL:** Apply the exact same analysis parameters (TFR calculation, clustering parameters, MVPA settings) used for the original data analysis (Steps 1–12) when analyzing the shuffled data. Any deviation in parameters will compromise the comparison and validation.
17. Interpret control analysis results.

Compare the outputs from the shuffled data analysis (dendrogram from Step 16.b.ii, decoding accuracy from Step 16.c.iii) with those obtained from the original data.

***Note:*** This comparison serves to validate the original findings. Typically, the expectation is that analysis of shuffled data will yield unstructured clustering and chance-level decoding performance, thereby increasing confidence in the results obtained from the original, structured data.

## Expected outcomes

This protocol integrates hierarchical clustering with multivariate pattern analysis (MVPA) to enhance the analysis of neural oscillations. First, hierarchical clustering provides an initial structure for frequency band classification, allowing for refinement beyond traditional fixed frequency bands. Second, the protocol employs MVPA to evaluate the functional relevance of these refined bands during task execution. By combining these complementary approaches, researchers can identify task-relevant frequency bands and quantify their contribution to neural information processing, gaining insights that might be overlooked by traditional methods.

**Figure 2:**
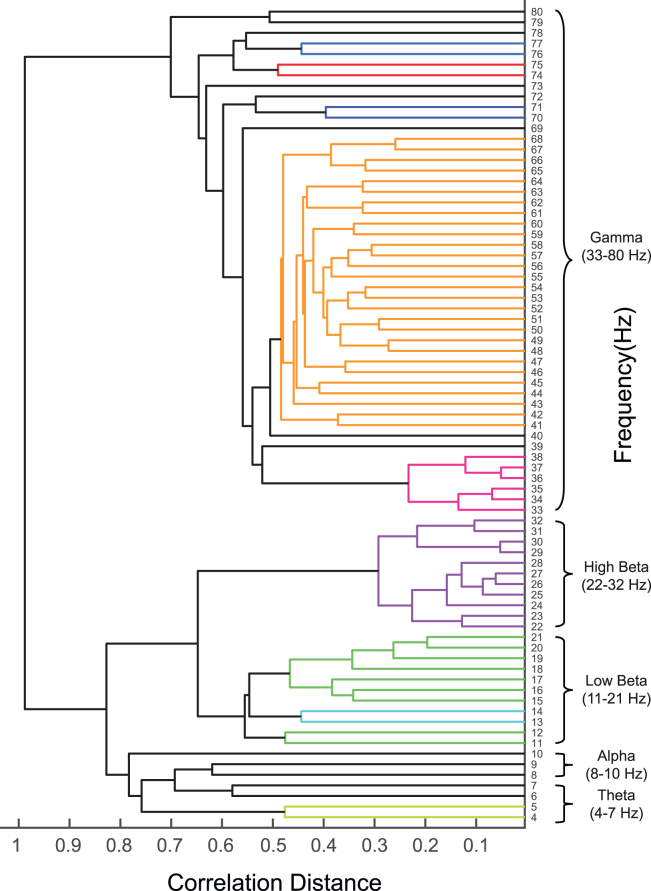
Example dendrogram for frequency band definition using hierarchical clustering Hierarchical clustering dendrogram derived from PFC ECoG signals. Frequency bins (y-axis) are grouped by the similarity of their temporal power profiles, measured by correlation distance (x-axis; smaller distance indicates higher similarity). Branch colors are visual aids automatically assigned by MATLAB. Brackets and labels illustrate an example of manually defined frequency bands (e.g., Theta, Alpha, Beta sub-bands, Gamma) resulting from user interpretation of the clustering structure. See also Figure S1.

Applying hierarchical clustering to electrocorticography (ECoG) data from the macaque prefrontal cortex (PFC) during a task involving passive viewing of visual stimuli consisting of equiluminant red, green, and blue figures (see our previous study^1^ for details) reveals distinct clusters of similar oscillatory components in the frequency domain, as depicted in Figure 2. This technique initially identifies potential frequency band clusters based on correlation distances between neural signals. The resulting data-informed structure can lead to final functional divisions (after manual refinement as described in ‘hierarchical clustering of time-frequency power’) that may differ from traditional classifications. Figure 2 illustrates that hierarchical clustering, as visualized in the dendrogram, can identify bands corresponding to theta, low alpha, high alpha/low beta, high beta, and gamma. While generally aligning with traditional frequency bands, these classifications also exhibit some variations, suggesting the potential for a more detailed and individualized frequency band structure compared to fixed ranges. Importantly, the strong correlation between the clustering results and established frequency bands highlights the efficacy of this initial clustering step in capturing the functional organization of neural oscillations and providing a solid foundation for subsequent refinement.

**Figure 3:**
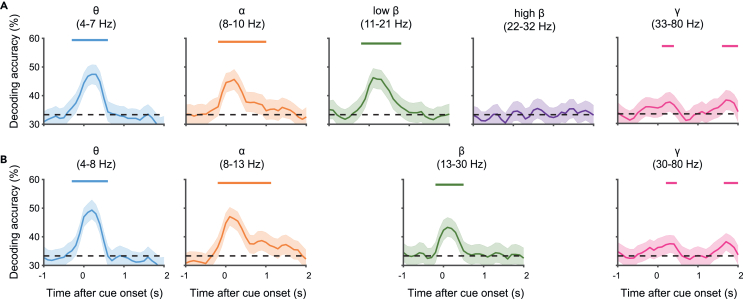
Comparison of MVPA decoding performance using different frequency band sets Time-series decoding accuracy (y-axis) for stimulus color in PFC ECoG data, plotted against time relative to stimulus onset (x-axis, seconds). Solid lines represent the median decoding accuracy, and shaded areas indicate the 95% confidence interval estimated via bootstrap resampling (*n* = 1000 iterations). Performance is compared using frequency bands derived from (A) the hierarchical clustering method presented in this protocol versus (B) conventional frequency band definitions. Colored lines above plots indicate time points with statistically significant decoding accuracy (*p* < 0.05, FDR corrected, bootstrap test, *n* = 1000 iterations). See also Figure S2.

Following the clustering process, we use MVPA to examine whether the neural oscillatory patterns in the PFC retain information about the passively perceived ‘color’. The decoder is trained to classify the color perceived by the monkey, using the power of each frequency band of the ECoG electrodes at a specific time as features. In this context, the decoding accuracy reflects the extent to which information about the observed color is encoded in the neural activity within a specific frequency band. The effectiveness of this approach is demonstrated by comparing the decoding results obtained using conventional frequency bands (e.g., alpha: 8–13 Hz, beta: 13–30 Hz)^18^ with those derived from hierarchical clustering (e.g., alpha: 8–10 Hz, low beta: 11–21 Hz, high beta: 22–32 Hz) (Figure 3). The bands derived from hierarchical clustering offer finer granularity and improved decoding accuracy, particularly within the 18–34 Hz range. In this range, the neural decoding accuracy is notably reduced, whereas conventional classifications group these activities within the broader beta band (13-30 Hz). This finding has practical implications for interpretation, suggesting that decoding performance broadly attributed to the conventional beta band may be primarily driven by activity within its lower frequencies (around 11–21 Hz in this case), with the upper frequencies (22–32 Hz) contributing less information relevant to this specific task. Such distinctions, revealed by refining frequency bands based on the data’s inherent structure, underscore the protocol’s utility in identifying more functionally specific oscillatory components that might be obscured by traditional, fixed band definitions.

**Figure 4:**
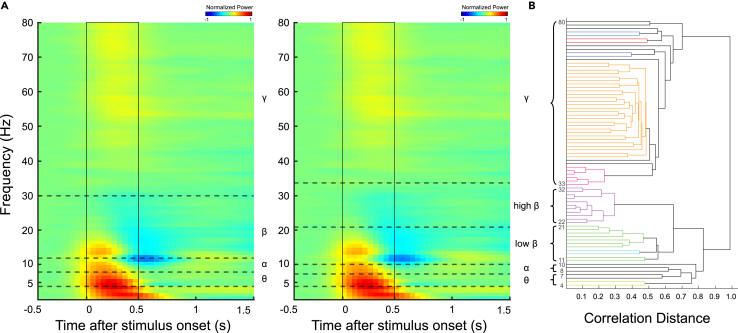
Visualization of conventional and hierarchically derived frequency band boundaries on the average TFR (A) The average TFR of normalized ECoG power in the PFC is shown in both panels (color indicates normalized power). Overlaid horizontal dashed lines illustrate different frequency band definitions: the left panel depicts conventional band boundaries, while the right panel shows example boundaries derived from hierarchical clustering. (B) The hierarchical clustering dendrogram (same as Figure 2) is shown alongside for reference to the bands used in panel (A), right.

This ability to discern potentially subtle differences in neural dynamics using the present protocol is further exemplified by the comparison in Figures 3 and 4. Specifically, the protocol allowed the differentiation of functionally distinct decoding performance within frequency ranges typically grouped together in conventional bands (e.g., the lower vs. upper parts of the beta band in Figure 3). Furthermore, as suggested visually in Figure 4, the boundaries derived from hierarchical clustering appear to align more closely with the actual dynamics of frequency power compared to fixed conventional boundaries. Revealing such functionally distinct sub-bands and achieving better alignment with power dynamics contribute to offering a more refined understanding of the temporal evolution of neural processing compared to analyses based solely on conventional approaches.

To further validate the protocol’s ability to identify meaningful neural patterns, we assessed its performance on control data lacking genuine temporal structure. Applying hierarchical clustering to temporally shuffled data, where time points within each trial and channel were randomized, revealed no consistent or neurophysiologically plausible frequency band organization, unlike the clear structure observed in the original data (Figure S1). This control demonstrates that the clustering method is unlikely to impose artificial band structures onto random noise. Furthermore, performing the MVPA decoding pipeline on this shuffled data, using frequency bands defined based on its unstructured dendrogram, resulted in decoding accuracies consistently at chance level across all time points and bands (Figure S2). This confirms the robustness of the decoding analysis and statistical evaluation, indicating that the significant decoding achieved on the original data reflects true task-related information rather than methodological artifacts.

## Limitations

While this protocol presents a valuable framework for analyzing neural oscillations by integrating hierarchical clustering and MVPA-based time-series decoding, certain limitations should be acknowledged for optimal implementation and interpretation.

A key limitation concerns the definition of frequency bands. Although hierarchical clustering provides a powerful data-informed starting point for identifying clusters in the frequency domain, the current protocol requires manual intervention to finalize the precise band boundaries. This refinement step typically involves visual inspection of the resulting dendrogram, often guided by consideration of established frequency band definitions or specific research hypotheses. The effectiveness of this combined approach lies in its practical utility: it allows researchers to leverage data-driven insights from clustering while retaining the flexibility to incorporate prior knowledge or address specific experimental questions, which purely automated methods might currently lack. However, this integration of data-driven clustering with experimenter judgment inevitably introduces a degree of subjectivity. Exploring and validating more automated methods for boundary determination based purely on the hierarchical clustering output could enhance reproducibility and objectivity in future iterations.

Furthermore, the frequency bands derived using this protocol may not always align perfectly with traditional frequency bands established in the literature. This potential discrepancy arises precisely because the method allows for refinement based on the data’s specific structure, which can reveal novel or more detailed frequency subdivisions not captured by fixed, conventional classifications. While identifying such potentially distinct bands is a strength of the approach, caution is necessary when interpreting bands that deviate significantly from established neurobiological categories. We strongly recommend validating the functional relevance of any novel or adjusted frequency bands using complementary methods, such as assessing their relationship with behavioral measures, task performance, or other established neurophysiological markers, to ensure they reflect meaningful neural processes rather than statistical idiosyncrasies.

Additionally, the optimal method for incorporating these potentially subject-specific frequency bands into group-level statistical analyses is not addressed in this protocol. Developing robust strategies to account for individual variability in band boundaries during group comparisons remains an important area for future methodological research.

The decoding accuracy achieved using this protocol, while informative, may vary depending on the specific dataset and experimental conditions. Factors such as signal quality (e.g., signal-to-noise ratio), the number of available trials, and the complexity of the task being decoded can significantly influence decoding performance. For instance, lower signal quality or fewer trials can reduce the statistical power to detect information encoded in neural activity, potentially leading to lower decoding accuracy. Conversely, highly complex tasks might require more sophisticated decoding models or feature representations than the simple band-power features used here. Careful consideration of these potential influences is crucial when interpreting the decoding results and comparing them across studies or conditions.

Finally, while the conceptual framework of combining hierarchical clustering and MVPA is broadly applicable, the specific implementation details and code provided are tailored for the MATLAB/FieldTrip/LibLinear/LIBSVM environment. Adapting this protocol for use with other software packages (e.g., EEGLAB, Brainstorm, Python libraries) would require significant user modifications to the code and potentially different parameter choices.

## Troubleshooting

### Problem 1

Insufficient memory (related to Steps 1 and 7).

### Potential solution

This protocol can require a significant amount of memory, especially when processing large datasets. If you encounter an "out of memory" error, consider increasing the available RAM on your computer or using a machine with more memory. You can also try reducing the memory demands by adjusting parameters in the TFR analysis. For example, in Steps 1 and 7, modifying the time of interest (toi) and frequency of interest (foi) parameters in the scripts (e.g., ‘P1_Averaging_TFR_div00.m‘ and ‘P3_Averaging_TFR_div01.m‘) to cover a smaller range or using coarser steps can reduce memory usage. Furthermore, you can utilize the ‘ft_selectdata’ function in the FieldTrip toolbox to select specific subsets of your data, such as particular channels or time segments, thereby reducing the overall data size loaded into memory. These adjustments can also lead to a reduction in the total analysis time.

### Problem 2

Program errors when using versions of MATLAB or the FieldTrip toolbox other than those we recommended (related to before you begin, Steps 7–11).

### Potential solution

MATLAB and the FieldTrip toolbox are frequently updated, and function specifications may change between versions. While we recommend using the versions specified in this protocol, if you choose to use other versions (especially newer ones), we suggest considering the following points based on our experience:

Newer versions of MATLAB: Errors may occur, particularly when saving figures or images. This is often due to frequent changes in MATLAB’s graphics and file-saving functions. Refer to the MathWorks online help for the specific MATLAB version you are using to find the correct function specifications and modify the code accordingly.

FieldTrip toolbox: The FieldTrip toolbox is updated even more frequently. It is important to note that newer versions are not always more stable. We recommend using the version specified in this protocol (fieldtrip-20240110), which is the latest version we have tested and confirmed to be working correctly.

### Problem 3

I want to run the analysis on a Mac or Linux machine with MATLAB installed (related to before you begin, Step 9).

### Potential solution

While we primarily recommend using Windows, we have confirmed that our code can run on macOS. Execution on Linux should also be possible in principle, but has not been systematically validated. Please be aware of the following points:

MEX files for SVM solvers: The SVM solvers we use for decoding analysis, LibLinear and LIBSVM, utilize MEX (MATLAB Executable) files for faster computation. While pre-compiled MEX files for Windows are included in the downloaded archives, you will need to compile the MEX files yourself for macOS and Linux. Instructions for compiling MEX files are typically found in the README file within the ‘matlab’ folder of the respective downloaded SVM solver’s toolbox folder.

Compatibility with M1 Macs and newer: If you are using a Mac with an M1 or newer Apple Silicon processor, older versions of MATLAB may not be able to compile MEX files properly. You may need to use a newer version of MATLAB (e.g., 2023b or later) that is specifically designed for Apple Silicon.

### Problem 4

Errors or warnings about the installation path of the FieldTrip toolbox occur when running scripts (related to before you begin, Step 8), such as:

Warning: Multiple versions of FieldTrip on your path will confound FieldTrip.

Warning: Your path is set up incorrectly. You probably used addpath(genpath('path_to_fieldtrip')), this can lead to unexpected behavior.

### Potential solution

These warnings indicate that MATLAB is having trouble locating the correct FieldTrip functions. This can happen if you have multiple versions of FieldTrip installed or if the path to FieldTrip is not set up correctly. Ensure that you have only one version of FieldTrip installed and that the path to this version is correctly added to your MATLAB path, as described in the "Software Requirements and Setup" section. When setting the path, it is recommended to remove paths to older or unnecessary versions of FieldTrip and use the ‘ft_defaults’ function as described in the official FieldTrip documentation (https://www.fieldtriptoolbox.org/faq/installation/).

### Problem 5

The frequency bands resulting from clustering seem unsuitable (too coarse or too fine) for the analysis goals (related to Steps 3 and 6).

### Potential solution

Selecting the best granularity requires user judgment based on the data and research goals. The script ‘P2_Hclust_freq_bands.m’ provides a starting point using a threshold (coph) calculated based on user-set ‘linkage_method’ and ‘cutoff_ratio’ parameters. Use these steps:

• Evaluate Initial Bands: Check the bands from initial settings. Consider:
  ○ Data Structure: Does the dendrogram support the divisions? Are bands consistent with TFR patterns?
  ○ Neurophysiological Context: Compare with conventional bands. Are they plausible?
  ○ Functional Relevance (Validation): Assess band performance using the independent MVPA results.
• Adjust if Suboptimal: If the initial bands lack validated functional relevance or clear data structure support:
  ○ Need Finer Bands? Try decreasing cutoff_ratio, or manually split bands.
  ○ Need Coarser Bands? Try increasing cutoff_ratio, or manually merge band.
  ○ Try Different Linkage? Change linkage_method, evaluating based on all criteria, prioritizing validated functional relevance.
• Iterate & Finalize: If iterating through parameters, the final choice should yield bands supported by data structure and demonstrating validated meaningful functional properties on the independent evaluation dataset.

### Problem 6

The statistical significance indicators are missing in the time-series decoding accuracy plot (related to Steps 9 and 11).

### Potential solution

This issue likely occurs when the statistical testing method is not consistently set between ’P4_TC_decoding.m’ and ’P5_TC_Plot.m’. Ensure that the ’flg_bootstrap’ parameter is set identically in both scripts. Additionally, when using the bootstrap test, confirm that ’ntimes_iter’ is set to a sufficiently large value. If applying FDR correction with the bootstrap test, a minimum of 1000 iterations is required to detect statistical significance. While a larger number of iterations generally leads to more precise p-value estimates, it also increases the computation time.

## Resource availability

### Lead contact

Further information and requests for resources and methods should be directed to and will be fulfilled by the lead contact, Hisashi Tanigawa (hisashi.q@gmail.com).

### Technical contact

Further technical and detailed inquiries should be directed to our technical contact, Chengpeng Li (chengpengli1997@hotmail.com).

### Materials availability

This study did not generate new unique reagents.

### Data and code availability

The code and sample data supporting the current study have been deposited to Zenodo (https://doi.org/10.5281/zenodo.14697222). Any additional information required to reanalyze the data reported in this paper is available from the lead contact upon request.

## Acknowledgments

This work was supported by grants from the National Natural Science Foundation of China (31872776) and the National Key R&D Program of China (2018YFA0701402) to H.T. and the 10.13039/501100001691Japan Society for the Promotion of Science (KAKENHI 23H00413) and 10.13039/100009619AMED grant (JP24wm0625205) to I.H.

## Author contributions

C.L. and H.T. compiled the example data based on data collected by Tanigawa et al.^1^ and Zhou et al.^7^ All authors wrote and revised the manuscript.

## Declaration of interests

The authors declare no competing interests.
