# Supplementary material for: Protocol for assisting frequency band definition and decoding neural dynamics using hierarchical clustering and multivariate pattern analysis
Source: STAR Protoc. 2025 Jun 3;6(2):103870. doi: 10.1016/j.xpro.2025.103870 (PMC12171811; doi:10.1016/j.xpro.2025.103870)
Supplement: Document S1. Figures S1 and S2 [file mmc1.pdf]

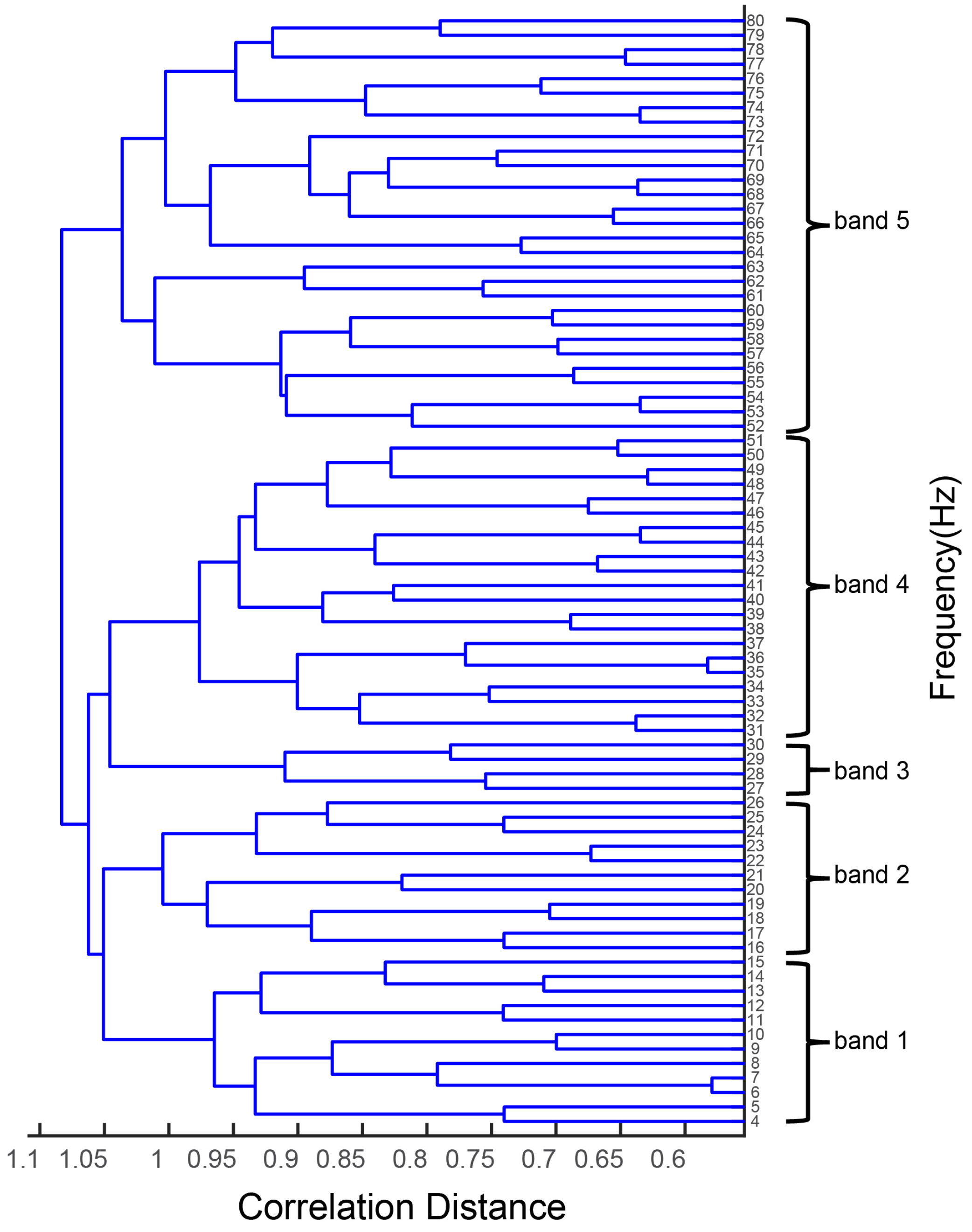

Figure S1. Hierarchical clustering dendrogram for temporally shuffled data, related to Part 7.

Result of applying the clustering analysis (Part 2) to control data generated by temporal shuffling (trldata_odd_shuffled.mat).

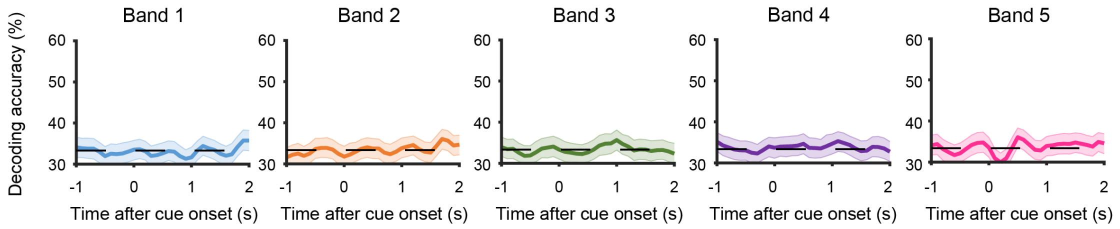

Figure S2. MVPA decoding accuracy for temporally shuffled data, related to Part 7.

Time course of decoding accuracy resulting from applying the MVPA pipeline (Parts 3-5) to temporally shuffled control data (trldata_even_shuffled.mat). Frequency bands were manually derived to approximate conventional bands from the dendrogram in Figure S1. Shaded areas represent 95% CI (bootstrap).
